# New survival standards for advanced melanoma

**DOI:** 10.1038/s41416-020-0738-5

**Published:** 2020-02-17

**Authors:** Lavinia Spain, James Larkin, Samra Turajlic

**Affiliations:** 1 Skin and Renal Unit, Royal Marsden NHS Foundation Trust, London, UK; 2 Department of Medical Oncology, Eastern Health, Melbourne, VIC Australia; 3 Eastern Health Clinical School, Monash University, Melbourne, VIC Australia; 4 Francis Crick Institute, London, UK

**Keywords:** Melanoma, Cancer immunotherapy

## Abstract

The expectation for survival in patients with advanced melanoma now exceeds 50% at 5 years in patients treated with first-line combination ipilimumab and nivolumab, despite this regimen being associated with substantial toxicity. We discuss the latest updates from the Checkmate-067 study, framing the role of this combination in practice today.

## Main

The most recent update of the Checkmate-067 trial has cemented the potential for durable, long-term survival in patients with advanced melanoma. Landmark survival rates at 5 years show that 52% of people treated with first-line combination ipilimumab and nivolumab (ipi + nivo), and 44% of those treated with nivolumab alone, are alive, in comparison with 26% treated with ipilimumab alone.^1^ In the Combi-D study evaluating first-line dabrafenib and trametinib, 34% of patients were alive at 5 years.^2^ The median OS has still not been reached for the whole cohort receiving ipi + nivo, and is 37 months for nivolumab alone. These results deliver a new ‘standard of survival’ for patients with advanced melanoma, and they set a high bar for other metastatic tumour types. Given the clear superiority of both nivolumab-containing regimens over ipilimumab monotherapy, they are the focus of further discussion in this Comment piece.

Not only are patients surviving for years despite advanced melanoma, many are experiencing durable remissions in the absence of further treatment. Around 20% of patients receiving ipi + nivo or nivolumab alone had a complete response to treatment, with ongoing responses at 5 years in 62% and 61%, respectively. Of those alive at 5 years, 74% who received ipi + nivo, and 58% who received nivolumab alone, had not required any subsequent systemic treatment. In the ipi + nivo arm, the median time from the last dose of study drug to the next line of systemic therapy was 18 months, whereas it was only 2 months for nivolumab alone.^1^ For many patients, not having to remain ‘on treatment’ comes as a huge advantage. Although formal data on the health economic advantages of this have not been published, one may hypothesise that these may favour the ipi + nivo regimen, given that a median of four cycles were received, versus 15 for nivolumab alone.^3^

Although this trial has never been powered for a formal comparison between the ipi + nivo and nivolumab monotherapy arms, there have been consistent subgroups in descriptive analyses who have emerged with favourable outcomes from the ipi + nivo arm, justifying the much greater rates of G3/4 toxicity (59% versus 24%). The updated descriptive analyses again suggest that ipi + nivo is probably superior to nivolumab alone in patients with an elevated lactate dehydrogenase (LDH) (38% versus 28% alive at 5 years, as opposed to 60% and 53% with a normal LDH), and in those with a BRAF mutation (60% versus 46% alive at 5 years, as opposed to 48% and 43% in the BRAF wild-type cohort), as previously mentioned.^3–5^ The benefit of ipi + nivo in patients with brain metastases has also been demonstrated in other studies.^6,7^ Programmed death ligand 1 (PD-L1), found to be heterogeneous in its expression in melanoma,^8^ again did not prove reliable as a sole biomarker. Although 5-year overall survival (OS) looked comparable between the nivolumab-containing regimens in patients with PD-L1 expression ≥1% (54% for ipi + nivo versus 52% for nivolumab alone), in patients with PD-L1 expression ≥10%, the ipi + nivo arm had a numerically higher rate of OS (59% versus 47%), although the numbers were relatively small.

One of the key deterrents of the ipi + nivo regimen for clinicians and patients is the higher rate of significant immune-related toxicity. The updated Checkmate-067 results provide reassurance for at least two reasons. Firstly, the 5-year OS rate for patients who stopped ipi + nivo during the induction phase (74 out of 315, 23%) was 51%, comparable with 52% in the overall intention-to-treat population, although median survival in this subgroup was reached (61.4 months). Secondly, novel long-term adverse events were not described.^1^ We await longer-term survival data from studies evaluating combination regimens with lower doses of ipilimumab^9,10^ to see whether these may be as effective without the same extent of toxicity. In keeping with this theme of reassurance, quality-of-life (QOL) analyses suggest that there is no meaningful deterioration in QOL on either ipi + nivo or nivolumab alone, during or after therapy. However, neither did the QOL analyses suggest any meaningful *improvement* from baseline. This may be due to the greater fitness of patients eligible for a clinical trial, as opposed to the common real-world scenario of treating patients who are heavily burdened by disease. The latter group may actually benefit from the higher rate of objective tumour shrinkage achieved with ipi + nivo (58% versus 45%).

While we now can debate whether 1 in 10 patients are actually cured with first-line ipi + nivo or nivolumab alone, we must also remember that half of the patients in this study had died by 5 years, highlighting a huge disparity in outcomes for patients with advanced melanoma. How can we close this gap? In addition to bringing these regimens earlier into the treatment pathway, and continuing to pursue novel immunotherapy combinations, what we really need is a multifaceted approach to evaluate the potential immune engagement between tumour and host prior to, and during, immune checkpoint therapy, and a way to personalise our strategy to the shortcomings. Heterogeneity between tumours within a patient underpinning mixed responses and strategies linked to liquid biopsy may be the most appropriate to represent a summary of tumour activity. Translating work in these areas into the clinic is a huge challenge and requires significant academic collaboration. In this era of two highly effective treatment classes for patients whose tumours possess a *BRAF V600* mutation, we are also faced with a lack of prospective evidence to inform our sequencing strategy, despite retrospective data to suggest that survival is favoured when immune checkpoint regimens are delivered prior to BRAF/MEK-targeted therapy.^11^ Whilst the glass is half-full for the outcome of many patients, we need to continue striving to meet the same standard for the rest.

## Data Availability

Not applicable.

## References

[1] Larkin J, Chiarion-Sileni V, Gonzalez R, Grob JJ, Rutkowski P, Lao CD (2019). Five-year survival with combined nivolumab and ipilimumab in advanced melanoma. N. Engl. J. Med.

[2] Robert C, Grob JJ, Stroyakovskiy D, Karaszewska B, Hauschild A, Levchenko E (2019). Five-year outcomes with dabrafenib plus trametinib in metastatic melanoma. N. Engl. J. Med.

[3] Larkin J, Chiarion-Sileni V, Gonzalez R, Grob JJ, Cowey CL, Lao CD (2015). Combined nivolumab and ipilimumab or monotherapy in untreated melanoma. N. Engl. J. Med.

[4] Wolchok JD, Chiarion-Sileni V, Gonzalez R, Rutkowski P, Grob JJ, Cowey CL (2017). Overall survival with combined nivolumab and ipilimumab in advanced melanoma. N. Engl. J. Med.

[5] Hodi FS, Chiarion-Sileni V, Gonzalez R, Grob JJ, Rutkowski P, Cowey CL (2018). Nivolumab plus ipilimumab or nivolumab alone versus ipilimumab alone in advanced melanoma (CheckMate 067): 4-year outcomes of a multicentre, randomised, phase 3 trial. Lancet Oncol..

[6] Tawbi HA, Forsyth PA, Algazi A, Hamid O, Hodi FS, Moschos SJ (2018). Combined nivolumab and ipilimumab in melanoma metastatic to the brain. N. Engl. J. Med.

[7] Long GV, Atkinson V, Lo S, Sandhu S, Guminski AD, Brown MP (2018). Combination nivolumab and ipilimumab or nivolumab alone in melanoma brain metastases: a multicentre randomised phase 2 study. Lancet Oncol..

[8] Sunshine JC, Nguyen PL, Kaunitz GJ, Cottrell TR, Berry S, Esandrio J (2017). PD-L1 expression in melanoma: a quantitative immunohistochemical antibody comparison. Clin. Cancer Res.

[9] Long GV, Atkinson V, Cebon JS, Jameson MB, Fitzharris BM, McNeil CM (2017). Standard-dose pembrolizumab in combination with reduced-dose ipilimumab for patients with advanced melanoma (KEYNOTE-029): an open-label, phase 1b trial. Lancet Oncol..

[10] Lebbe C, Meyer N, Mortier L, Marquez-Rodas I, Robert C, Rutkowski P (2019). Evaluation of two dosing regimens for nivolumab in combination with ipilimumab in patients with advanced melanoma: results from the phase IIIb/IV checkmate 511 trial. J. Clin. Oncol..

[11] Mason R., Dearden H. C., Nguyen B., Oon J. S., Smith J. L., Randhawa M., et al. Combined ipilimumab and nivolumab first-line and after BRAF-targeted therapy in advanced melanoma. *Pigment Cell Melanoma Res*. 2019. 10.1111/pcmr.12831. [Epub ahead of print] 10.1111/pcmr.12831 31587511

